# Identification of copy number alterations in colon cancer from analysis of amplicon-based next generation sequencing data

**DOI:** 10.18632/oncotarget.24912

**Published:** 2018-04-17

**Authors:** Duarte Mendes Oliveira, Gianluca Santamaria, Carmelo Laudanna, Simona Migliozzi, Pietro Zoppoli, Michael Quist, Catie Grasso, Chiara Mignogna, Laura Elia, Maria Concetta Faniello, Cinzia Marinaro, Rosario Sacco, Francesco Corcione, Giuseppe Viglietto, Donatella Malanga, Antonia Rizzuto

**Affiliations:** ^1^ Dipartimento di Medicina Sperimentale e Clinica, Università Magna Graecia, Catanzaro, Italy; ^2^ Dipartimento di Scienze della Salute, Università Magna Graecia, Catanzaro, Italy; ^3^ Dipartimento di Scienze Mediche e Chirurgiche, Università Magna Graecia, Catanzaro, Italy; ^4^ Fred Hutchinson Cancer Research Center, Seattle, WA, USA; ^5^ University of California Los Angeles (UCLA), Los Angeles, CA, USA; ^6^ UOC Chirurgia Generale, Azienda Ospedaliera dei Colli, Napoli, Italy

**Keywords:** colon cancer, Next generation sequencing, copy number alteration

## Abstract

The objective of this study was to determine the feasibility to detect copy number alterations in colon cancer samples using Next Generation Sequencing data and to elucidate the association between copy number alterations in specific genes and the development of cancer in different colon segments. We report the successful detection of somatic changes in gene copy number in 37 colon cancer patients by analysis of sequencing data through Amplicon CNA Algorithm. Overall, we have found a total of 748 significant copy number alterations in 230 significant genes, of which 143 showed CN losses and 87 showed CN gains. Validation of results was performed on 20 representative genes by quantitative qPCR and/or immunostaining. By this analysis, we have identified 4 genes that were subjected to copy number alterations in tumors arising in all colon segments (defined “common genes”) and the presence of copy number alterations in 14 genes that were significantly associated to one specific site (defined “site-associated genes”). Finally, copy number alterations in ASXL1, TSC1 and IL7R turned out to be clinically relevant since the loss of TSC1 and IL7R was associated with advanced stages and/or reduced survival whereas copy number gain of ASXL1 was associated with good prognosis.

## INTRODUCTION

The development of cancer is driven by the acquisition of somatic genetic alterations that include single nucleotide variations (SNVs), gene fusions and copy-number alterations (CNAs). CNAs are somatic changes that cause the gain or loss of DNA fragments [1–3], and represent the most common alterations of cancer cells [4–9]. They contribute to both onset and progression of cancer by inappropriate activation of proto-oncogenes and/or inactivation of tumor suppressor genes [4, 10–15]. Characterization of CNAs in tumors have helped in the identification of relevant oncogenes including ERBB2 and EGFR, as well as tumor suppressors such as pRB and TP53 [16], resulting into better diagnostics and more appropriate therapy [17–19].

Recent studies revealed that development of Colon Cancer (CC) involves stepwise accumulation of CNAs [20–23]. CC is the second most commonly diagnosed cancer in females and the third in males, with over 50,000 new cancer patients in Italy every year [24]. CC is a heterogeneous disease that displays a characteristic molecular stepwise progression [25]. Numerous studies have reported on the identification of somatic CNAs in CC [26–37], which, in some cases, have been associated to clinical outcome or metastatic progression [38–42]. However, many of these studies had inherent limitations due to small sample size, low-resolution assays and/or lack of associated clinical annotation, particularly for early-stage CC.

This notwithstanding, previous studies have established that the most frequent CNAs in CC are CN gains at chromosomes 7p, 7q, 13q, 20p, 20q, Xp and Xq and CN losses at chromosomes 8p, 17p, 18p and 18q.27 [16]. Notably, colon adenomas apparently have similar levels of CNAs as carcinomas [43, 44] whereas the highest levels of CNAs were detected in metastatic CC [39]. All these studies have led to the identification of multiple oncogenes (EGFR, ERBB2, CCND1, MET, MYC) and/or tumor suppressors (TP53, APC, SMAD4) [45, 46].

Previous studies of CNAs detection in CC were carried out by quantitative PCR, fluorescence *in situ* hybridization (FISH), whole-genome array comparative genomic hybridization (aCGH) or single-nucleotide polymorphism (SNP) arrays [47–49]. The recent advent of high-throughput sequencing techniques and the subsequent development of *ad hoc* algorithms have made available CNAs identification [50, 51]. Recently, Grasso and co-workers have developed an algorithm for assessing CNAs from Next Generation Sequencing (NGS) data generated by amplicon-based DNA libraries derived from Formalin Fixed Paraffin-Embedded (FFPE) tumors [52].

In the present study, we applied this algorithm to DNA sequencing data to determine the feasibility to detect CNAs using NGS and elucidate the association between specific CNAs and cancer originating from different anatomical colon segments.

## RESULTS

### Identification of CNAs in colon cancer by analysis of amplicon-based NGS data

**Figure 1:**
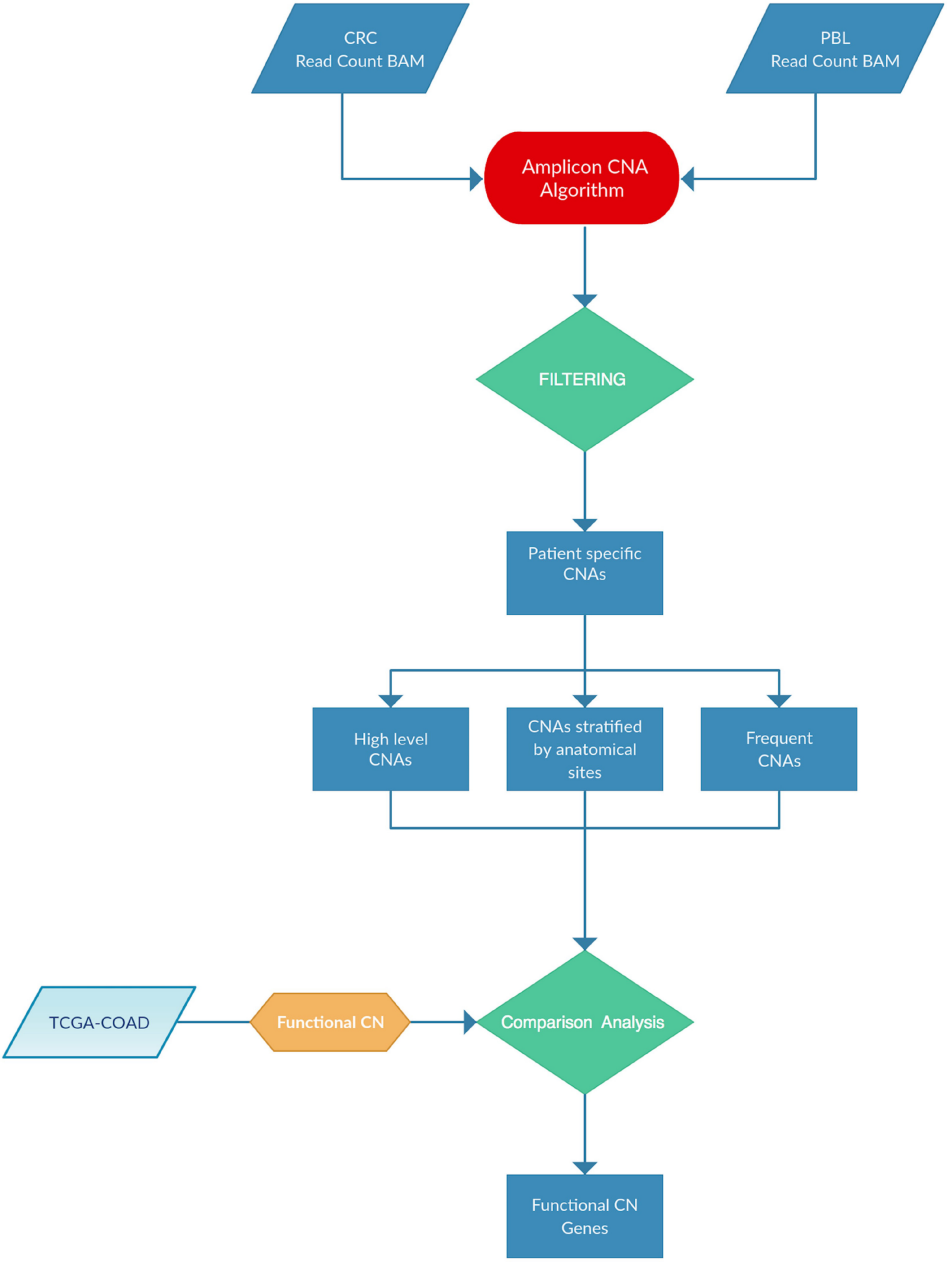
Pipeline analysis implemented for this study

In this manuscript we have applied the Amplicon CNA Algorithm, previously described by Grasso and co-workers [52] to identify somatic CNAs from amplicon-based NGS data generated using the Ion AmpliSeq™ Comprehensive Cancer Panel (CCP), which provides complete whole exon coverage of the 409 most important cancer-associated genes. As described in a parallel manuscript (Oliveira *et al.*, under revision), we have selected 37 patients among those who underwent surgery for CC at the General Surgery Unit of University Magna Graecia of Catanzaro in the years 2013–2015. The samples were resected from multiple anatomical segments of the colon: ascending colon (7 patients), descending colon (7 patients), hepatic flexure (8 patients), splenic flexure (5 patients), transverse colon (4 patients) and cecum (6 patients) (Supplementary Figure 1). Complete demographic and clinical information of patients are reported in Supplementary Table 1. NGS was performed in 37 tumor samples and 13 matched PBLs (Oliveira *et al.*, under revision). Figure 1 illustrates the pipeline analysis of NGS data implemented in R statistical environment [53]. The input for the Amplicon CNA Algorithm was the Binary Alignment Multifasta (BAM) file. The use of pooled normal samples as reference has been reported to be a valid alternative to the one-by-one match between tumors and the corresponding normal tissues [52]. The algorithm output consisted in a list of all CNAs identified for each sample analyzed. Overall, 1904 CNA calls were identified. Copy Number (CN) gains were defined as alterations showing log2 CN ratio ≥ 0.1 and CN losses were defined as alterations showing log2 CN ratio ≤ –0.1. Benjamini-Hochberg procedure was used to reduce false discovery rate and CNAs were considered significant when the *q*-value was ≤ 0.05 (Supplementary File 1).

**Figure 2:**
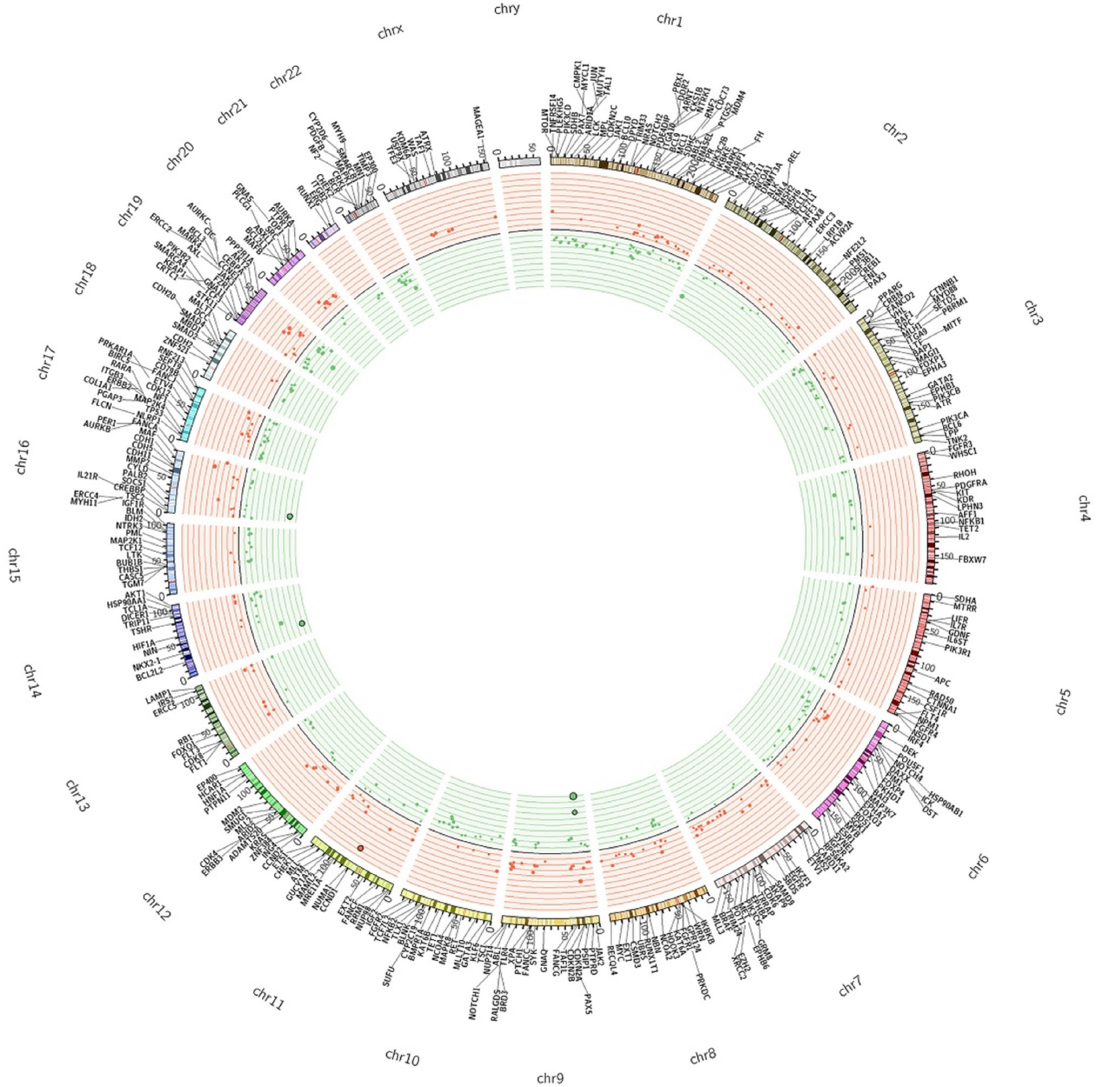
The Circos plot summarizes all CNAs detected in colon cancers samples under analysis The two outermost tracks report the distributions of 409 genes along the genome; the innermost tracks reported the values of the log2 CN ratio. Genes with altered CN are distinguished by colour as deletions (green) and amplifications (red).

**Figure 3:**
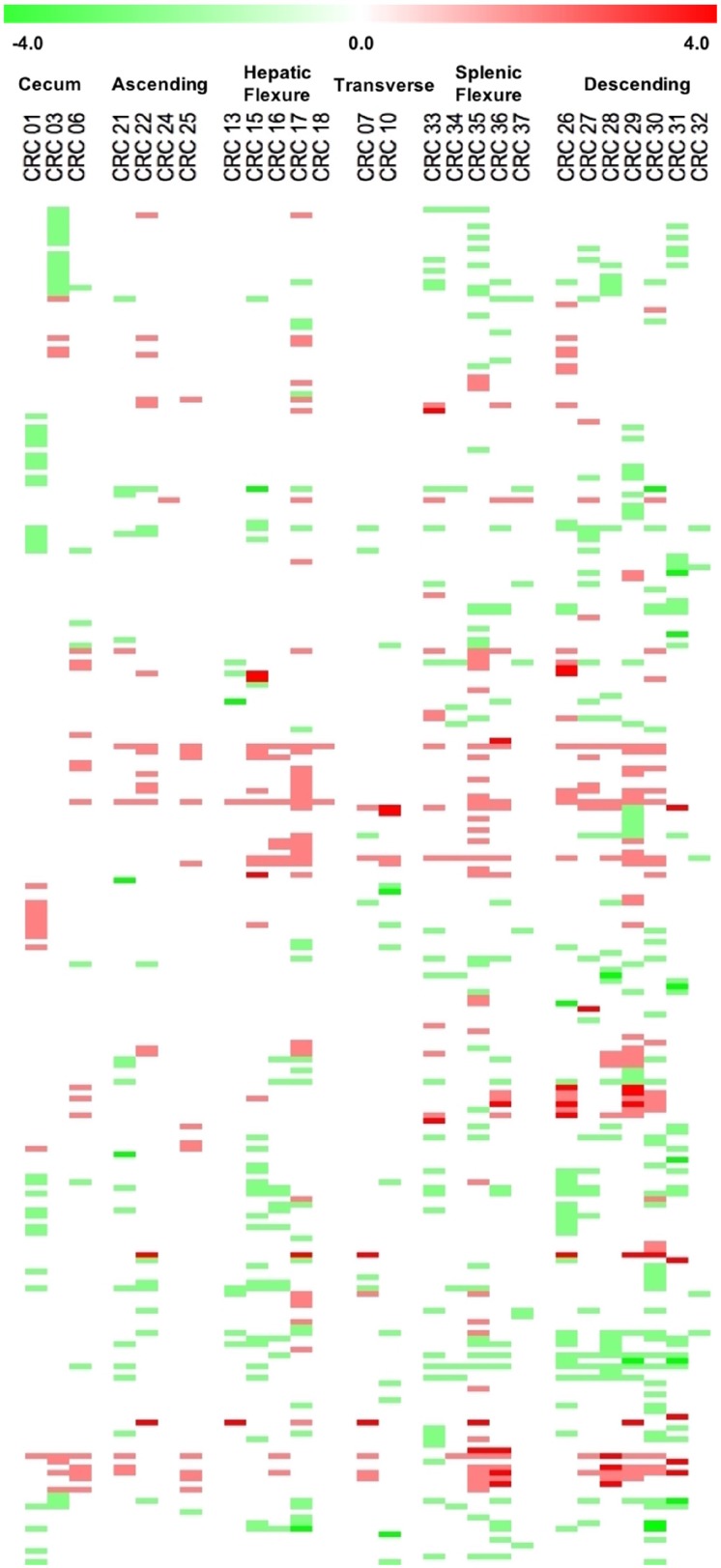
Heatmap representation of the data across all the cytobands harbouring CNAs ordered by chromosome position Colour intensity is proportional to degree of CNAs, one-copy gains are indicated in light red and one-copy losses are indicated in light green.

On the basis of these parameters we found a total of 785 significant CNA calls, of which 328 (41.7%) were CN gains and 457 (58.2%) were CN losses, distributed along 243 different genes. Finally, for the sake of clarity, we considered significant only the alteration calls that showed concordance in ≥65% of the samples. Using this threshold, we found that the majority of genes with CNAs were concordant (230/243). The remaining 13 genes (DICER1, FGFR1, HOOK3, IGF2, IKBKB, MARK1, NFKB1, NF1, PTPRD, SMAD2, SYNE1, TAF1L, TRIP11) presented discordant calls and were not considered in the rest of the manuscript. We will consider only the 230 genes that showed concordant calls. Among the concordant genes, 143 presented CN losses and 87 presented CN gains (Supplementary Table 2). The Circos plot shown in Figure 2 summarizes all detected CNAs. The median number of genes per tumor showing CN gains was 12 (range 1–37) whereas the median number of genes per tumor showing CN losses was 20 (range 1–41). CN gains and losses involved genes located on all chromosomes except chromosome 23. The heatmap in Figure 3 shows all significant CNAs ordered by cytobands (see also Supplementary Table 3), with CN gains (red) and CN losses (green).

As indicated in Table 1 (columns 1–8), the most frequent CN gains were observed at chromosomes 7, 20 and 8. Frequent CN gains involved MLL3 at chromosome 7q36.1 (*n* = 16, 43.24%), AKAP9 at chromosome 7q21.2 (*n* = 15, 40.54%), ASXL1 at chromosome 20q11.21 (*n* = 14, 37.84%), PLCG1 at chromosome 20q12 (*n* = 13, 35.14%), and UBR5 at chromosome 8q22.3 (*n* = 13, 35.14%).

**Table 1:** Most frequently detected CNAs and corresponding values in TCGA

CNAs DETECTED IN 37 CC								TCGA			
Gene symbol	Chromosome band	Number of samples showing CNA	Percentage of samples showing CNA	Log2 (CNA)*	Mean CN ratio **	Range of CN ratio	Type of CNA	Percentage of samples showing CN GAIN	Number of samples showing CN GAIN	Percentage of samples showing CN LOSS	Number of samples showing CN LOSS
MLL3	7q36.1	16	43.24	0.41	1.34	1.14–1.49	GAIN	47.21	169	1.96	7
AKAP9	7q21.2	15	40.54	0.47	1.4	1.21–1.52	GAIN	47.49	170	1.12	4
ASXL1	20q11.21	14	37.84	0.72	1.66	1.40–2.10	GAIN	70.95	254	0	0
UBR5	8q22.3	13	35.14	0.54	1.47	1.23–1.72	GAIN	55.87	200	1.96	7
PLCG1	20q12	13	35.14	0.89	1.88	1.39–2.55	GAIN	71.23	255	0.56	2
KAT6A	8p11.21	9	24.32	0.79	1.79	1.43–3.22	GAIN	42.74	153	9.5	34
DST	6p12.1	8	21.62	0.36	1.29	1.17–1.42	GAIN	19.27	69	8.38	30
ATR	3q23	7	18.92	0.49	1.41	1.19–1.54	GAIN	14.53	52	7.82	28
BRAF	7q34	7	18.92	0.66	1.58	1.41–1.67	GAIN	47.77	171	1.12	4
CSMD3	8q23.3	7	18.92	0.53	1.47	1.29–1.92	GAIN	55.87	200	2.51	9
DCC	18q21.2	13	35.14	-0.61	0.66	0.54–0.81	LOSS	2.23	8	61.17	219
WHSC1	4p16.3	12	32.43	-0.50	0.71	0.63–0.75	LOSS	2.23	8	27.65	99
NLRP1	17p13.2	10	27.03	-0.60	0.66	0.55–0.76	LOSS	3.63	13	53.07	190
MBD1	18q21.1	10	27.03	-0.67	0.63	0.54–0.72	LOSS	2.79	10	59.22	212
EP400	12q24.33	9	24.32	-0.51	0.7	0.56–0.76	LOSS	18.99	68	12.01	43
TGM7	15q15.2	9	24.32	-0.71	0.61	0.50–0.72	LOSS	2.79	10	37.43	134
RNF213	17q25.3	9	24.32	-0.36	0.78	0.71–0.86	LOSS	23.46	84	12.01	43
ERG	21q22.2	9	24.32	-0.67	0.63	0.48–0.78	LOSS	4.75	17	27.93	100
GATA2	3q21.3	8	21.62	-0.85	0.56	0.45–0.65	LOSS	13.97	50	7.54	27
LTK	15q15.1	8	21.62	-0.64	0.64	0.55–0.72	LOSS	2.79	10	37.43	134

^*^Base 2 logarithm of CN ratio.

^**^Ratio of the Tumor Vs Normal intensity signal.

Conversely, the most frequent CN losses were MBD1 at 18q21.1 (*n* = 10, 27%), DCC at 18q21.2 (*n* = 13, 35.14%), WHSC1 (*n* = 12, 32.43%) at 4p16.3, NLRP1 (*n* = 10, 27%) and RNF213 (*n* = 9, 24.3%) at 17p13.2 and 17q25.3, respectively.

Other genes that, in this study, presented frequent CN gains were KAT6A at 8p11.21, CSMD3 at 8q23.3, PTPRT at 20q12, DST at 6p12.1 and ATR at 3q23. Conversely frequent CN losses were observed for TGM7 at 15q15.2, EP400 at 12q24.33, CDH2 at 18q12.1, LTK at 15q15.1 and ERG at 21q22.2.

In Table 2 are listed the genes with the highest values of CN changes (columns 1–8). Among these, there were CCND1 with a mean CN ratio of 6.0, CCNE1 with a mean CN ratio of 4, MAF with a mean CN ratio of 3.30, BCL2L1 with a mean CN ratio of 2.19 and CDKN2A with a mean CN ratio of 0.2.

**Table 2:** Genes showing high level of CN alteration and corresponding values in TCGA

CNAs DETECTED IN 37 CC								TCGA			
Gene symbol	Chromosome band	Number of samples showing CNA	Percentage of samples showing CNA	Log2 (CNA)^*^	Mean CN Ratio^**^	Range of CN ratio	Type of CNA	Percentage of samples showing CN GAIN	Number of samples showing CN GAIN	Percentage of samples showing CN LOSS	Number of samples showing CN LOSS
CCND1	11q13.3	1	2.70	2.58	6	5.99–5.99	GAIN	41	11.45	51	14.25
CCNE1	19q12	1	2.70	2.03	4.09	4.09–4.09	GAIN	72	20.11	25	6.98
MAF	16q23.2	6	16.21	1.52	2.92	2.48–4.16	GAIN	84	23.46	23	6.42
BCL2L1	20q11.21	2	5.40	1.41	2.66	2.63–2.68	GAIN	254	70.95	0	0
SRC	20q11.23	2	5.40	1.39	2.92	1.62–4.21	GAIN	255	71.23	1	0.28
IRS2	13q34	1	2.70	1.35	2.55	2.54–2.54	GAIN	211	58.94	7	1.96
CEBPA	19q13.11	6	16.21	1.31	2.53	1.95–3.54	GAIN	69	19.27	25	6.98
PMS2	7p22.1	1	2.70	1.11	2.16	2.15–2.15	GAIN	195	54.47	1	0.28
FANCA	16q24.3	1	2.70	1.11	2.15	2.15–2.15	GAIN	85	23.74	27	7.54
CDK8	13q12.13	3	8.10	1.09	2.18	1.57–2.69	GAIN	214	59.78	8	2.23
CDKN2A	9p21.3	1	2.70	−2.13	0.23	0.22–0.22	LOSS	60	16.76	48	13.41
NPM1	5q35.1	1	2.70	−1.26	0.42	0.41–0.41	LOSS	24	6.7	67	18.72
TCL1A	14q32.13	1	2.70	−1.24	0.42	0.42–0.42	LOSS	25	6.98	118	32.96
CHEK2	22q12.1	1	2.70	−1.19	0.44	0.43–0.43	LOSS	9	2.51	117	32.68
PAX5	9p13.2	1	2.70	−1.17	0.45	0.44–0.44	LOSS	59	16.48	41	11.45
SUFU	10q24.32	1	2.70	−1.07	0.48	0.47–0.47	LOSS	12	3.35	80	22.35
HSP90AA1	14q32.31	1	2.70	−1.03	0.49	0.48–0.48	LOSS	24	6.7	118	32.96
FOXO3	6q21	3	8.10	−1	0.5	0.47–0.51	LOSS	51	14.25	49	13.69
NRAS	1p13.2	1	2.70	−0.99	0.5	0.50–0.50	LOSS	15	4.19	88	24.58
FBXW7	4q31.3	2	5.40	−0.94	0.52	0.49–0.55	LOSS	13	3.63	101	28.21

^*^Base 2 logarithm of CN ratio.

^**^Ratio of the Tumor Vs Normal intensity signal.

Expectedly, most genes showing alterations had already been reported to be associated with the development of colon cancer [22]. However, among the 230 genes that presented significant CN changes in colon cancer we identified at least 10 whose alterations had not been previously associated to colon cancer. Of these 4 genes were subjected to CN gains (DST, KLF6, FANCA, CSMD3) and 6 were subjected to CN losses (TGM7, NKX2-1, RHOH, RNF213, ERG, and CRBN).

### Validation of NGS results by Q-PCR analysis and immunohistochemistry analysis

**Figure 4:**
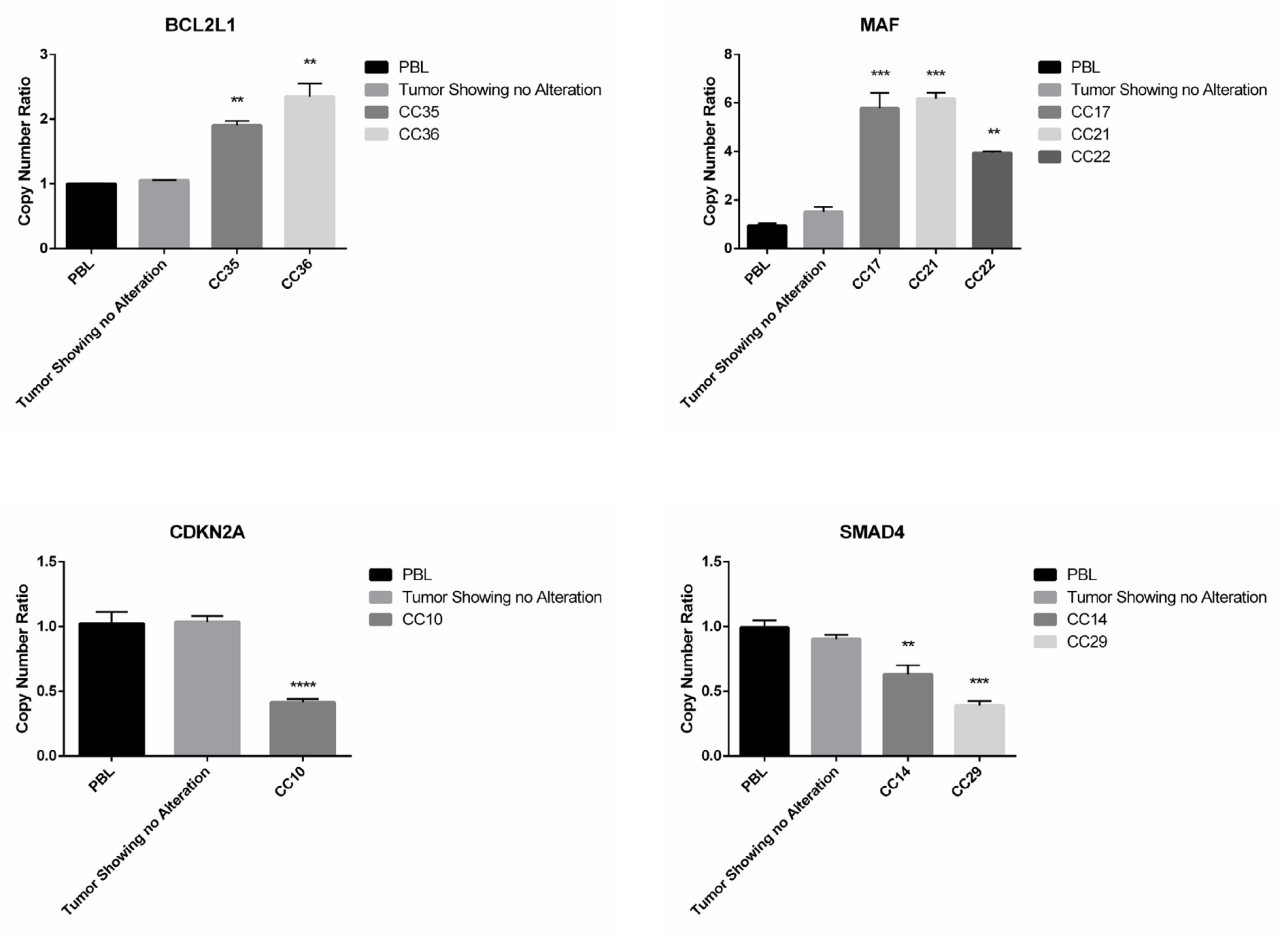
Q-PCR analysis of samples presenting CNAs in SMAD4, CDKN2A, MAF and BCL2L1 Q-RT-PCR analysis of CN losses in SMAD4 and CDKN2A and of CN gains in MAF and BCL2L1. Values are expressed as CN ratio using as standard the median value of 3 PBL samples set as 1. Tumors presenting normal CN for the specific genes were also included in each experiment. Statistical significance as indicated when confronted with PBL.

**Figure 5:**
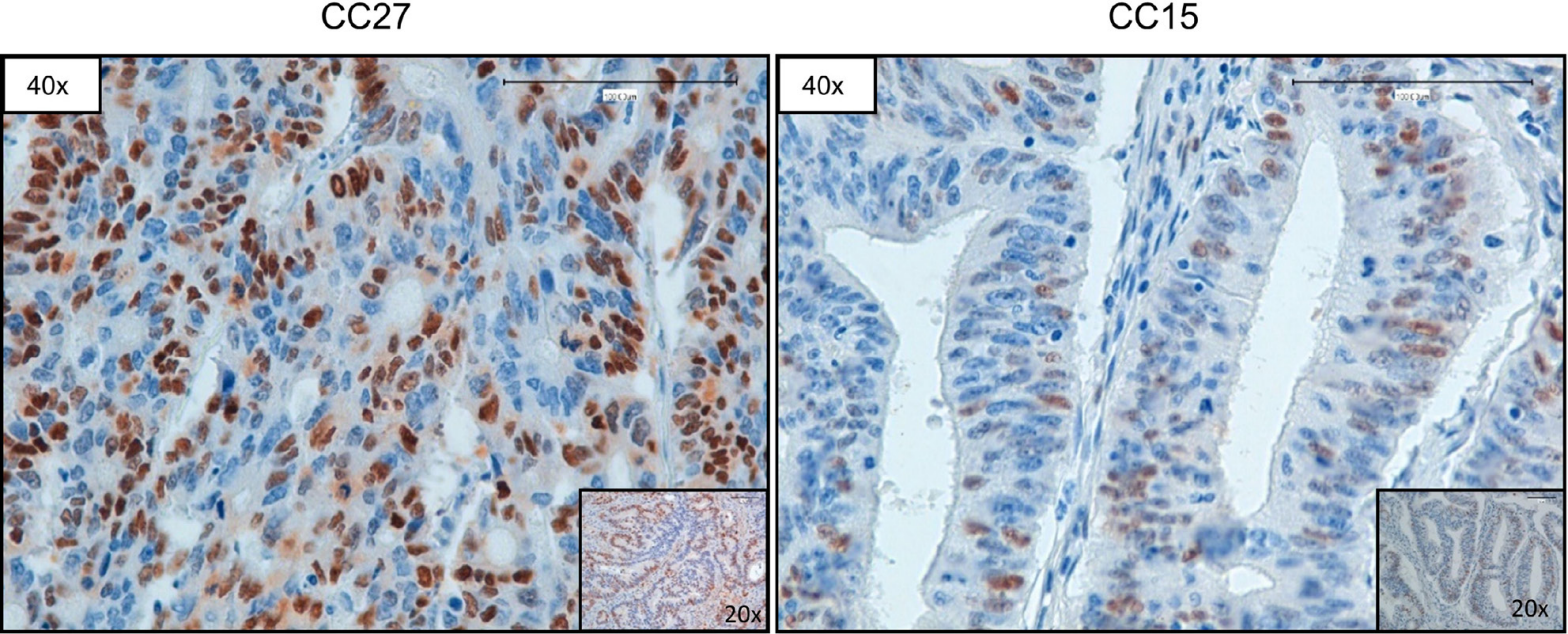
Representative images of CCND1 immunostaining Sample CC27 (with CN gain of CCND1) and sample CC15 (with normal CN of CCND1), respectively. Magnification as indicated.

Subsequently, we used Q-PCR to validate the results obtained through the bioinformatics analysis of NGS data. In Figure 4 we reported representative Q-PCR analysis relative to 2 genes showing CN gains (MAF and BCL2L1) and 2 genes with CN losses (SMAD4, CDKN2A). In Supplementary Figure 2 we reported representative Q-PCR relative to additional genes showing significant CNVs. Overall Q-PCR results were consistent with bioinformatics analysis of NGS data, also in those genes that resulted discordant (Supplementary Figure 2). Immunostaining analysis demonstrated that the observed CNA in the gene encoding CCND1 resulted into cyclin D1 protein overexpression in CC27 (see Figure 5).

Comparison of the results with Colon Adenocarcinoma dataset present in public repositories

We validated the results obtained in this study relative to the association of genes with CNAs and localization of tumors in colon segments by analysing the Colon Adenocarcinoma dataset within The Cancer Genome Atlas database (COAD-TCGA). Within this dataset, 358 tumor samples arising from different colon segments were present. Eighty tumors derived from ascending colon, 146 from descending colon, 6 from splenic flexure, 16 from hepatic flexure, 84 from cecum and 27 from transverse colon.

**Figure 6:**
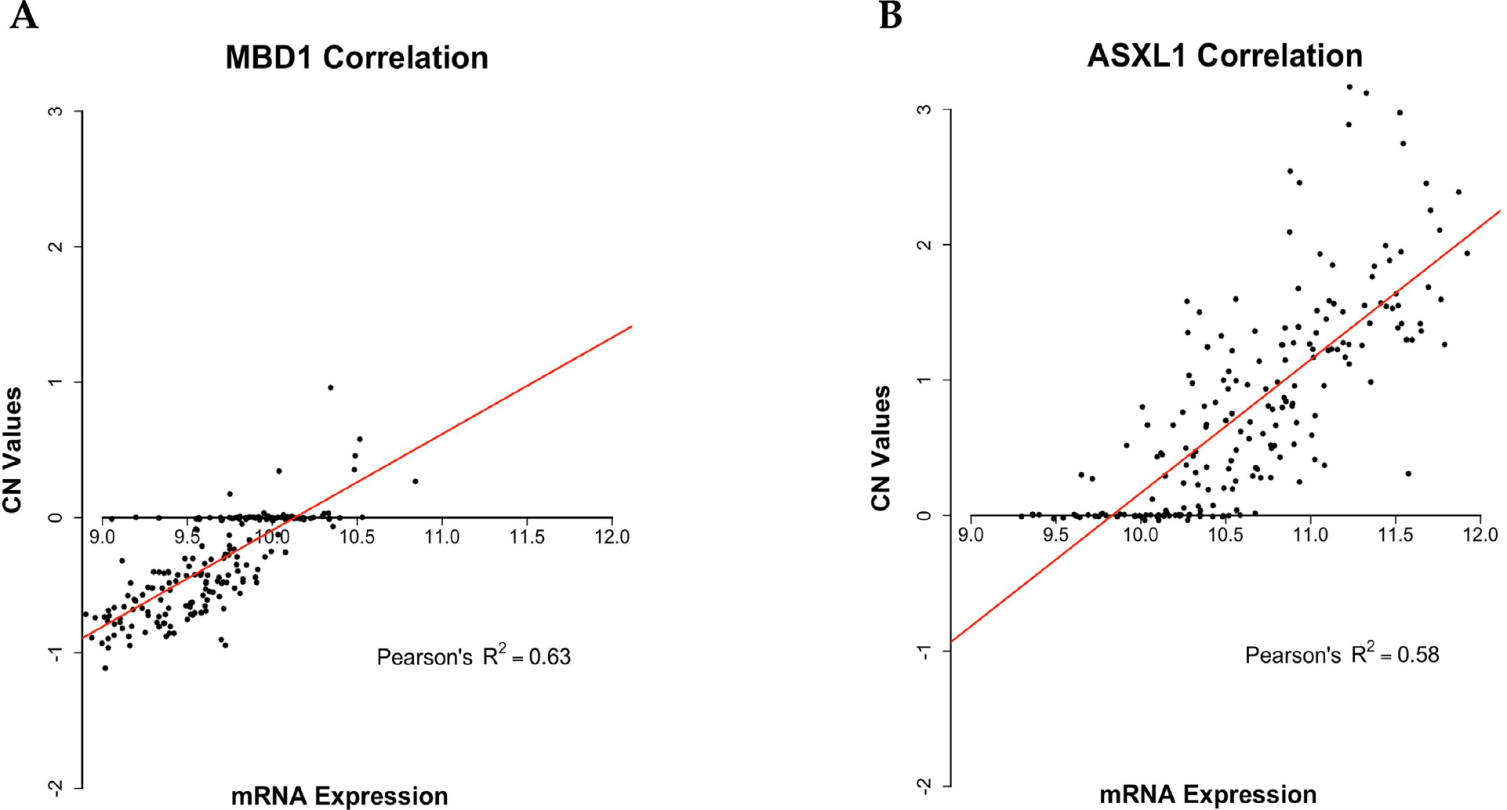
Scatter plots representing Pearson correlation between CN and mRNA expression Scatter plots showing Pearson correlation between CN and mRNA expression as downloaded from the COAD-TCGA dataset. (**A**) Pearson correlation between CN and mRNA expression of MBD1 (*R*^2^ = 0.62). (**B**) Pearson correlation between CN and mRNA expression of ASXL1 (R^2^ = 0.58). Linear regression lines are represented in red, CN value = 0 represent diploid status.

We further validated the analysis of CNAs by investigating the correlation between the 230 genes showing CNAs identified in this study and the corresponding mRNA expression reported in the COAD-TCGA dataset. The analysis was performed by a linear regression model, which allowed the identification of genes presenting direct association between CN changes (i.e. amplification/loss) and RNA expression (i.e. over-expression/under-expression). Among the identified 230 genes with significant CNAs, 69 genes (30%) showed a statistically significant Pearson correlation value higher than 0.45 (*R*^2^ > 0.2, *p*-value < 0.01). See Supplementary Table 4 for further details. Notably, some among the 69 genes with a significant correlation between CN changes and RNA expression are involved in the development of CC, such as SMAD2, ASXL1, PLCG1, UBR5, TOP1 and MBD1. In Figure 6 are reported two of the genes that showed the most significant correlation between CNAs and mRNA expression (ASXL1 and MBD1).

### Clinical-pathological correlations

For clinical analysis we selected those genes that presented concordant CNAs in at least 5% of the samples. We found that 60, among 230 significant genes, presented CNAs in ≥ 5% of patients and correlated them with clinical and pathological parameters such as node status (N), stage, tumor size (T) and/or presence of metastasis (M1) (*p*-value < 0.05). See Supplementary Table 5. Expectedly, Univariate Cox Regression analysis demonstrated that the parameters T, M and stage were predictors of overall survival (OS) (see Table 3).

**Table 3:** Univariate Cox regression analysis of OS of CC patients, genes showing CNAs and the clinical covariates previously selected by Log-Rank test

Covariates	OS		
	HR	95% CI	*p*-value
T (TMN) (2–3/4)	11.2	1.16–108	0.008^*^
Stage (I + II + III/IV)	0.08	0.013–0.563	0.0022^*^
M (TNM) stage (M0/M1)	11.37	1.77–72.8	0.002^*^
TSC1	5.8	0.85–39.38	0.05^*^
IL7R	5.8	0.85–39.39	0.05^*^
ASXL1	1.62	0.25–10.52	0.04^*^

^*^p-value ≤ 0.05.

**Figure 7:**
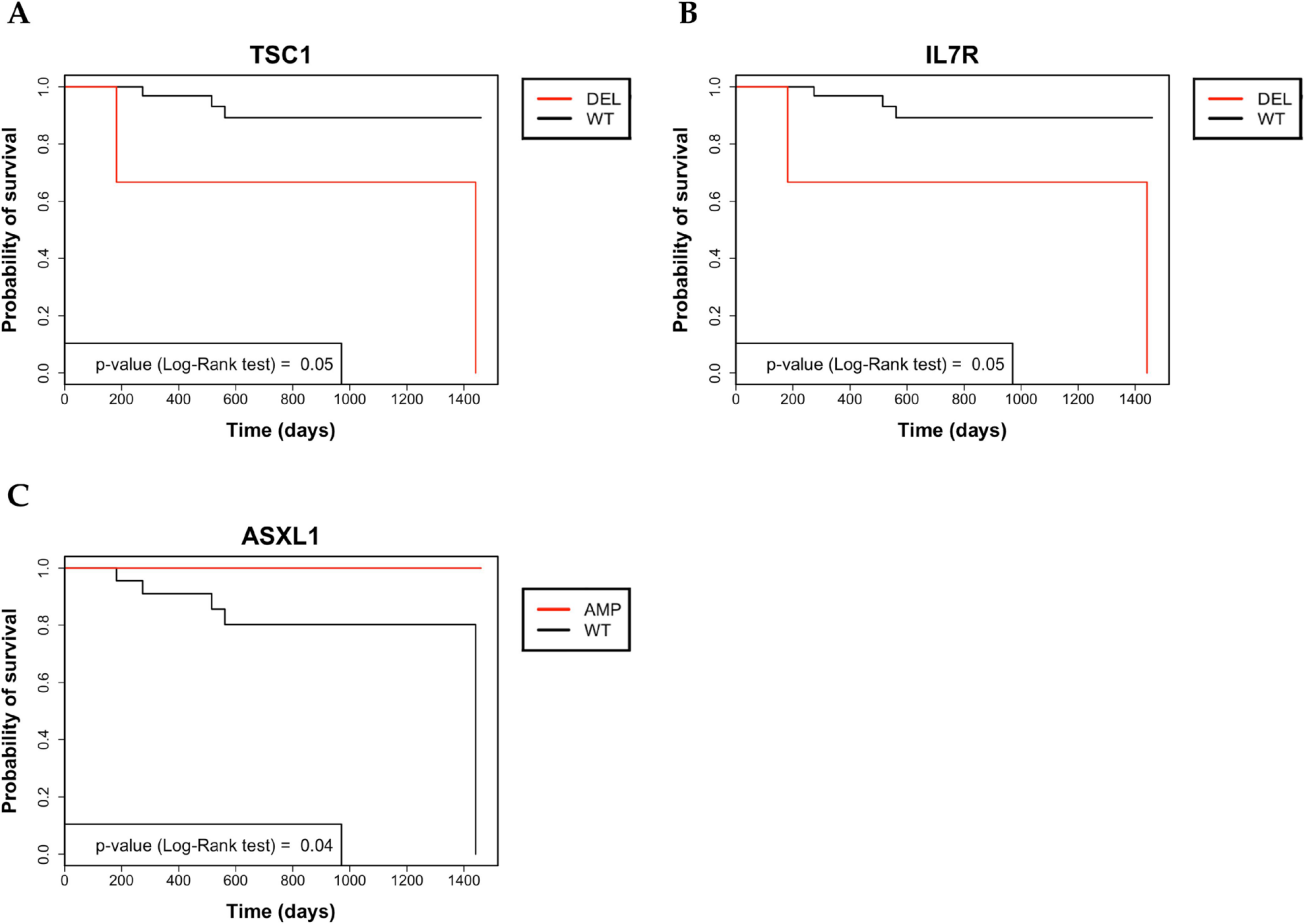
Kaplan–Meier analysis of 4-year survival in colon cancer patients (**A**) Kaplan–Meier survival curve of OS in in colon cancer patients that were stratified for being positive or negative for CNAs in TSC1. (**B**) Kaplan–Meier survival curve of OS in in colon cancer patients that were stratified for being positive or negative for CNAs in IL7R. (**C**) Kaplan–Meier survival curve of OS in colon cancer patients that were stratified for being positive or negative for CNAs in ASXL1.

Subsequently, the genetic status of CC patients (*n* = 35) was correlated with OS and the gain of ASXL1, loss of TSC1 or loss of IL7R predicted poor prognosis, as shown by the corresponding Kaplan–Meier curves (see Figure 7).

In particular, the average 4-year survival rate of all CC patients was 86%. Upon stratification for the status of ASXL1, TSC1 or IL7R genes, the 4-year survival rates of CNA-negative patients were 73.3% for ASXL1, 91% for TSC1 and 91% for IL7R. Conversely, the 4-year survival rates of patients showing gain of ASXL1, loss of TSC1 and loss of IL7R were 100%, 33.3% and 33.3%, respectively. Patients with CN gain in ASXL1 showed a mean survival time of 48 months with no dead among the 14 patients under analysis. Conversely, ASXL1-negative patients showed mean survival of 41, with 5 dead patients out of 22.

Patients with CN loss in TSC1 (2 dead patients out of 3) showed mean survival of 34 months whereas patients with no TSC1 alterations (3 dead patients out of 33) showed mean survival of 45 months. Patients with CN loss in IL7R (2 dead patients out of 3) showed mean survival time of 34 months in comparison with patients showing normal genetic status of IL7R, mean survival of 45 months (3 dead patients out of 33).

However, none of the covariates that resulted significant in Univariate analysis turned out to be independent prognostic factors by Multivariate Cox Regression analysis.

### Genes showing CNAs in colon cancer arising in different anatomical segments

In order to identify a specific pattern of genetic alterations based on tumor site, we stratified genes with CN alterations according to the anatomical localization of the tumor (ascending colon, descending colon, transverse colon, hepatic flexure, splenic flexure and cecum). Genes that presented gains or losses in all colon segments were defined “common genes”. Genes that presented gains or losses significantly associated to one anatomic colon segment were defined “site-associated genes”. “Common genes” and “site-associated genes” are listed in Supplementary Tables 6 and Table 4, respectively.

**Table 4:** Genes with CNAs showing significant association with tumors arising in specific colon sites by χ2 test

GENE SYMBOL		*p*-value	Anatomic Site					
			Ascending colon (*n* = 7)	Cecum (*n* = 6)	Descending colon (*n* = 7)	Hepatic Flexure (*n* = 8)	Splenic Flexure (*n* = 5)	Transverse colon (*n* = 4)
**Genes** **with CN LOSS**	**MBD1**	0.0005	Obs^*^ = 0 Exp^**^= 0.75 (10.7%)	Obs = 0 Exp = 0.64 (10.7%)	Obs = 6 (85.7%) Exp = 0.75 (10.7%)	Obs = 1 (12.5%) Exp = 0.86 (10.7%)	Obs = 3 (60%) Exp = 0.54 (10.7%)	Obs = 0 Exp = 0.43 (10.7%)
	**PDGFB**	0.0008	Obs = 0 Exp = 0.56 (8%)	Obs = 0 Exp = 0.48 (8%)	Obs = 0 Exp = 0.56 (8%)	Obs = 0 Exp = 0.64 (8%)	Obs = 3 (60%) Exp = 0.4 (8%)	Obs = 0 Exp = 0.32 (8%)
	**DCC**	0.002	Obs = 1 (14.28%) Exp = 2.45 (35%)	Obs = 1 (16.66%) Exp = 2.1 (35%)	Obs = 6 (85.71%) Exp = 2.45 (35%)	Obs = 1 (12.5%) Exp = 2.81 (35%)	Obs = 4 (80%) Exp = 1.75 (35%)	Obs = 0 Exp = 1.4 (35%)
	**WRN**	0.007	Obs = 0 Exp = 0.94 (13%)	Obs = 0 Exp = 0.81 (13%)	Obs = 4 (57.14%) Exp = 0.94 (13%)	Obs = 0 Exp = 1.08 (13%)	Obs = 0 Exp = 0.67 (13%)	Obs = 1 (25%) Exp = 0.54 (13%)
	**NOTCH2**	0.007	Obs = 0 Exp = 0.75 (11%)	Obs = 1 (16.66%) Exp = 0.64 (11%)	Obs = 0 Exp = 0.75 (11%)	Obs = 0 Exp = 0.86 (11%)	Obs = 3 (60%) Exp = 0.64 (11%)	Obs = 0 Exp = 0.43 (11%)
	**SMAD4**	0.01	Obs = 0 Exp = 0.56 (8%)	Obs = 0 Exp = 0.48 (8%)	Obs = 3 (42.85%) Exp = 0.56 (8%)	Obs = 0 Exp = 0.64 (8%)	Obs = 0 Exp = 0.4 (8%)	Obs = 0 Exp = 0.32 (8%)
	**RNF213**	0.03	Obs = 1 (14.28%) Exp = 1.7 (24%)	Obs = 0 Exp = 1.45 (24%)	Obs = 5 (71.42) Exp = 1.7 (24%)	Obs = 2 (25%) Exp = 1.94 (24%)	Obs = 0 Exp = 1.21 (24%)	Obs = 1 (25%) Exp = 0.97 (24%)
	**APC**	0.03	Obs = 0 Exp = 0.94 (13 %)	Obs = 0 Exp = 0.81 (13 %)	Obs = 3 (42.85%) Exp = 0.94 (13 %)	Obs = 0 Exp = 1.08 (13 %)	Obs = 2 (40%) Exp = 0.67 (13 %)	Obs = 0 Exp = 0.54 (13 %)
	**PIK3R1**	0.03	Obs = 0 Exp = 0.94 (13%)	Obs = 0 Exp = 0.81 (13 %)	Obs = 3 (42.85%) Exp = 0.94 (13 %)	Obs = 0 Exp = 1.08 (13 %)	Obs = 2 (40%) Exp = 0.67 (13 %)	Obs = 0 Exp = 0.54 (13 %)
	**RET**	0.04	Obs = 0 Exp = 1.13 (16%)	Obs = 0 Exp = 0.97 (16%)	Obs = 2 (28.57%) Exp = 1.13 (16%)	Obs = 1 (12.5%) Exp = 1.29 (16%)	Obs = 3 (60%) Exp = 0.81 (16%)	Obs = 0 Exp = 0.64 (16%)
	**PKHD1**	0.04	Obs = 0 Exp = 1.13 (16%)	Obs = 0 Exp = 0.97 (16%)	Obs = 2 (28.57%) Exp = 1.13 (16%)	Obs = 1 (12.5%) Exp = 1.29 (16%)	Obs = 3 (60%) Exp = 0.81 (16%)	Obs = 0 Exp = 0.64 (16%)
**Genes with CN GAIN**	**RB1**	0.01	Obs = 0 Exp = 0.56 (8%)	Obs = 0 Exp = 0.48 (8%)	Obs = 3 (42.85%) Exp = 0.56 (8%)	Obs = 0 Exp = 0.64 (8%)	Obs = 0 Exp = 0.4 (8%)	Obs = 0 Exp = 0.32 (8%)
	**BCL2L1**	0.01	Obs = 0 Exp = 0.37 (5%)	Obs = 0 Exp = 0.32 (5%)	Obs = 0 Exp = 0.37 (5%)	Obs = 0 Exp = 0.43 (5%)	Obs = 2 (40%) Exp = 0.27 (5%)	Obs = 0 Exp = 0.21 (5%)
	**UBR5**	0.02	Obs = 0 Exp = 2.45 (35%)	Obs = 0 Exp = 2.1 (5%)	Obs = 4 (57.14%) Exp = 2.45 (5%)	Obs = 3 (37.5%) Exp = 2.81 (5%)	Obs = 4 (80%) Exp = 1.75 (5%)	Obs = 2 (50%) Exp = 1.4 (5%)

^*^Obs: Observed CC patients showing CNAs.

^**^Exp: Expected CC patients showing CNAs.

Analysis of the results demonstrated that tumors in the ascending colon presented 49 genes with CNAs (26 gains and 23 losses) with a mean value of 14.25 CNAs/tumor (1–24); Tumors arising in the descending colon presented 169 genes with CNAs (53 gains and 108 losses, 8 discordants) with a mean value of 40.85 CNAs/tumor (range 4–61); tumors arising in transverse colon presented 23 genes with CNAs (8 gains and 15 losses) with a mean value of 12.5 CNAs/tumor (range 10–15); tumors in the hepatic flexure presented 90 genes with CNAs (40 gains and 48 losses, 2 discordant) with a mean value of 24.8 CNAs/tumor (range 2–61); tumors in the splenic flexure presented 111 genes with CNAs (46 gains and 62 losses, 3 discordant) with a mean value of 32.8 CNAs/tumor (range 9–65); and tumors in the cecum presented 75 genes with CNAs (28 gains and 47 losses) with a mean value of 26.6 CNAs/tumor (range 20–36). See circos plots in Supplementary Figures 3-8.

Among “common genes”, CN gains were observed in PLCG1 and ASXL1 genes, whereas CN losses were observed in NLRP1 and WHSC1 genes.

“Site-associated genes” were identified by use of a chi-square test with a threshold of significance set at *p*-value ≤ 0.05. As listed in Table 4, “site-associated genes” that showed significant association with tumors arising in specific colon sites were 14. Among these, CN losses were identified in APC, DCC, MBD1, NOTCH2, PDGFB, PKHD1, PIK3R1, RET, RNF213, SMAD4 and WRN whereas CN gains were identified in BCL2L1, RB1 and UBR5.

Among the 14 “site-associated genes” that showed the most significant association with colon segments, PDGFB, SMAD4, RB1, BCL2L1 showed anatomical position dependency for only one site with highly significant *p*-values (≤ 0.01). CN loss of PDGFB was observed only in tumors from splenic flexure, CN loss of SMAD4 was observed only in tumors from descending colon, whereas CN gain of RB1 was observed only in tumors from descending colon and CN gain of BCL2L1 was observed only in tumors from splenic flexure.

On the other hand, CN loss of WRN, NOTCH2, APC and PIK3R1 were observed in tumors arising in two colon segments. WRN was lost in descending colon tumors (57%) and transverse colon tumors (25%); NOTCH2 was lost in tumors arising in splenic flexure (60%) and cecum (17%); APC was lost in tumors arising in descending colon (43%) and splenic flexure (40%); PIK3R1 was lost in tumors of descending colon (43%) and splenic flexure (40%).

Finally, PKHD1, RET, MDB1 RNF213, DCC and UBR5 showed less specific dependency on anatomical position. PKHD1 and RET were lost predominantly in splenic flexure tumors and descending colon tumors (60% and 29%, respectively) and less frequently in hepatic flexure tumors (12.5%); MDB1 was lost predominantly in tumors from ascending colon and splenic flexure (85% and 60%, respectively) and less frequently in hepatic flexure tumors (12.5%); RNF213 was lost predominantly in descending colon tumors (71%) but also in tumors from hepatic flexure, transverse colon and ascending colon (25%, 25%, 14%); DCC was lost in tumors from 5 different segments: splenic flexure, descending colon, ascending colon, cecum and hepatic flexure (80%, 86%, 14% 17%, 12.5%); UBR5 showed CN gain in tumors from splenic flexure, descending colon, transverse colon, and hepatic flexure (80%, 57%, 50% and 37.5%, respectively).

Given the limited number of samples analysed in the cohort of patients under study here, the significance of the anatomical position dependency shown for the 14 “site-associated genes” described above was investigated using a larger dataset of CC (COAD-TCGA). Notably, we found a significant association for 7 of the “site-associated genes” reported in this study also for samples present in COAD-TCGA (*n* = 358). In particular, CNAs in MBD1, SMAD4, PIK3R1, DCC, WRN, RB1 were significantly associated with tumors originating in descending colon whereas CNAs in NOTCH2 was associated with tumors originating in splenic flexure (*p*-value < 0.05).

These findings confirmed the significance of the anatomical position dependency shown for at least 7 out of 14 “site-associated genes” reported in this study, indicating that tumors arising in different colon segments may be caused by alterations that occur in different genes.

## DISCUSSION

In this manuscript, we have applied a previously described Amplicon CNA Algorithm, to investigate the presence of somatic CNAs in tumors originating in different colon sites [52]. This analysis was performed on NGS data generated using the Ion AmpliSeq™ CCP, which provides full exon coverage of the 409 most important cancer-associated genes. The main results reported in this manuscript were: i) the successful detection of somatic CNAs in 230 genes from NGS amplicon-based libraries in CC samples (143 genes with CN losses and 87 genes with CN gains), ii) the identification of clinically relevant CNAs in genes such as ASXL1, TSC1 and IL7R, iii) the identification of CNAs in 4 genes in tumors originating from all colon segments (“common genes”) and iv) the detection of CNAs in 14 genes associated preferentially to a specific colon site (“site-associated genes”).

The main characteristic of the approach described by Grasso *et al.*, and applied here, was to use read counts/amplicon to identify CNAs from NGS data. Prediction of gene amplification/deletion is possible if sufficient number of amplicons is analyzed [54]. Overall, we have found a total of 785 significant CNAs in 243 different genes, of which 328 were CN gains and 457 were CN losses. The results obtained *in silico* with the Amplicon CNA Algorithm were validated by quantitative Q-PCR and immunostaining. Further control of our results was performed by combining information from COAD-TCGA database and sequencing data presented in this manuscript. From this analysis it appears that almost 30% of the 230 genes with significant CNAs in CC showed a statistically significant correlation with mRNA expression, at difference with a similar analysis using the COAD-TCGA dataset, in which 20% of genes (3542 out of 17630) presented a positive correlation between CNAs and mRNA expression (*R*^2^ > 0.2; *p* ≤ 0.01).

Previous studies have reported that in colon cancer chr20 was most frequently subjected to CN gains and chr18 was most frequently subjected to CN losses [55]. In agreement with these previous reports, the genes most frequently subjected to CN gains observed in this study were located on chromosome 20 including ASXL1, PLCG1, TOP1 and PTPRT whereas the genes most frequently subjected to CN losses were located on chromosome 18 and include MBD1, DCC and CDH2. Most of the genes that presented CNAs identified in this manuscript, such as TOP1, ASXL1, PTPRD, DCC, NLRP1 and CDH2 have already been directly associated with the development of CC. In particular, gene amplification and/or overexpression of TOP1 has been detected in metastatic colon cancer whereas loss of ASXL1 occurs in CC with microsatellite instability [56]. Loss of PTPRD expression was observed in highly invasive cancers and correlated with patient survival [57].

Some of the genes that presented the highest values of CNAs have already been associated with CC. In particular, gene amplification and/or overexpression of CCND1 have been associated with poor prognosis and reduced overall survival in CC patients whereas BCL2L1 has been shown to play a role in the adenoma-to-carcinoma progression [58, 59]. Less clear is the role of MAF having been described either as an oncogene or as a tumor suppressor, depending on the cell context [60].

On the other hand, at least 10, among the 230 genes showing CNAs, were not known to be associated to CC. Among these TGM7, NKX2-1, RHOH, RNF213, ERG, and CRBN presented significant CN losses and thus were potential tumor suppressor genes whereas DST, KLF6, FANCA, CSMD3 presented significant CN gains and can be considered potential oncogenes.

Notably, we observed that CN gain of ASXL1 was associated with an improvement in OS, whereas CN loss of TSC1 and IL7R predicted significantly reduced OS.

An important aim of this study was to determine whether tumors arising in different colon segments presented specific molecular alterations. Overall we have identified 4 “common genes” subjected to CNAs in tumors originating from all colon segments and 14 “site-associated genes” whose alterations are associated to tumors arising in specific colon segments. Among the 4 “common genes” we found CN gains in ASXL1 and PLGC1, which suggest that they act as oncogenes in CC. However previous studies showing the involvement of ASXL1 and PLGC1 in the development of CC were inconsistent. In fact both ASXL1 and PLGC1 have been shown to act either as tumor suppressor genes or oncogenes [56, 61]. Among the “common genes” presenting CN losses identified in this study is NLRP1, a protein whose function is apparently involved in gastrointestinal inflammation and tumorigenesis [62].

Among the 14 “site-associated genes” that showed highly significant association with a specific colon segment, PDGFB, SMAD4, RB1, BCL2L1 showed anatomical position dependency for only one colon segment, WRN, NOTCH2, APC and PIK3R1 showed anatomical position dependency for two colon segments whereas the remaining genes PKHD1, RET, MDB1 RNF213, UBR5 and DCC were associated to 3 or more segments. Moreover, a further support to the significance of the association reported in this study, was the finding that 7 out of the 14 “site-associated genes” reported here showed a significant position dependency also in the cohort of tumors present within the COAD-TCGA database. In particular, CNAs in MBD1, SMAD4, PIK3R1, DCC, WRN, RB1 were significantly associated with tumors originating in descending colon whereas CNAs in NOTCH2 was associated with tumors originating in splenic flexure.

It is also of note that loss of “site-associated” genes such as WRN, NOTCH2, MBD1 and PI3KR1 had already been associated to development of human cancer. In particular, WRN has been reported to be frequently deleted in CC, and its deficiency apparently predisposes to various types of cancer [22]. In the present study WNR was found to be lost preferentially in descending colon tumors. Similarly, in agreement with the existing literature [63], we found that NOTCH2 was deleted in CC (preferentially in splenic flexure tumors), which suggested a role as tumor suppressor in this subset of CC. On the other hand, we have found that MBD1 loss is a frequent event in colon carcinogenesis, being associated with descending colon tumors in different studies. This observation is in agreement with previous studies reporting frequent deletion of 18q21 (where MBD1 maps) in CC [64]. Notably, the results described in the present study show a significant association between MBD1 loss and late stage of disease. Finally, loss of PIK3R1 was preferentially observed in descending colon tumors. PIK3R1 represents the p85 regulatory subunit of heterodimeric enzymes, the PI3Ks, which also include a p110 catalytic subunit [65]. PI3Ks are downstream effectors of tyrosine kinase and G-protein-coupled receptors, which coordinate multiple cell functions including proliferation, migration and survival [66, 67]. PIK3R1 plays an important role in restraining cell migration. Loss of PIK3R1 was observed in patients with stage III disease (5/12 patients) but in none of the patients with stage I or II disease (0/20), suggesting that its down-regulation promotes aberrant activation of PI3K signalling in colon cancer cells, which would lead to invasion of adjacent tissues and/or regional dissemination.

In conclusion, in this study we report the successful detection of somatic CNAs in 230 genes using NGS data relative to 37 CC samples. Expectedly, most genes showing CNAs had already been reported to be associated with CC [22] whereas at least 10 among the 230 altered genes had not apparently been associated to CC yet. Notably, the analysis reported in this study indicated that CN changes in at least 3 genes (ASXL1, TSC1 and IL7R) were clinically relevant, being their alteration associated with survival. Finally, the analysis of the distribution of genes with CNAs relative to the site of origin of cancer led to the identification of 4 “common genes” that were subjected to CNAs in tumors arising in all 6 colon segments and 14 “site-associated genes” whose CNAs occurs preferentially in tumors originating only in certain colon segments.

## MATERIALS AND METHODS

### Ethics statement

Accrual of patients was conducted according to Institutional Review Board of the AOU Mater Domini/University Magna Graecia (Catanzaro, Italy). The study was approved by the Institutional Review Board of the AOU Mater Domini/University Magna Graecia in the meeting of May 21st, 2014.

### Tumor samples

Tumor samples, matched normal mucosa and peripheral blood lymphocytes (PBL) were obtained from patients referring to General Surgery unit of the AOU Mater Domini/University Magna Graecia (Catanzaro, Italy), who underwent surgical resection for colon cancer since January 2013. Biopsies were immediately snap frozen and stored at –80° C. Hematoxylin and eosin-stained tissue sections were reviewed by an expert pathologist to confirm diagnosis.

### Patients’ demographics

General demographic information, histo-patological and clinical parameters, surgical treatment and follow-up data were collected prospectively and are also reported in Oliveira *et al.* (submitted). However, for sake of clarity we summarize below the clinical characteristics of the patients included in the study.

Among the 37 patients, 13 were women and 24 were males. Mean age of patients was 68.35 years old (range 47–84). Stage was known for 36 of the 37 patients: 7 patients had stage I disease, 13 patients had stage II disease, 12 patients had stage III disease and 4 patients had stage IV disease. Grade was known for 35 out of 37 patients: 1 patient had tumor that was graded G1, 25 patients had tumors that were graded G2 and 9 patients had tumors that were graded G3. Of the patients included in the present study, four presented distant metastasis. None of the patients received chemotherapy or radiation therapy prior to surgery.

### Bioinformatic analysis for CNV detection

DNA extraction, library preparation using the Ion AmpliSeq^™^ Comprehensive Cancer Panel on the Ion Torrent platform (Thermofisher, MA, USA), sequencing and NGS primary analysis were carried out as described (Oliveira *et al.*, submitted).

To identify CNAs in NGS amplicon-based dataset we replaced average coverage of exon pull-down regions with read counts per amplicon. All reads were aligned to the human reference genome (hg19). For each sample, normalization was performed by dividing the number of reads of each amplicon by the total number of reads. Subsequently, the normalized reads obtained as described from tumor samples were divided by the normalized reads from pool made of blood samples from 13 patients, set as reference. The resulting Log2 values (raw copy number ratios) were corrected for the GC content in each amplicon and the Poisson model was applied using the CNA amplicon algorithm described in Grasso *et al.* [52] to identify CNAs. Genes were defined significant when the *q*-value was ≤ 0.05 after the Benjamini-Hochberg correction [68]. CN gains were defined as genes showing log2 CN ratio ≥ 0.1 and CN losses were defined as genes showing log2 CN ratio ≤ −0.1.

### Association and survival studies

The association between genes showing CNAs and the clinical-pathological parameters was evaluated by Fisher’s exact test and χ^2^ test. Overall survival (OS) was calculated from the day of surgery to death or end of follow-up. Kaplan–Meier curves were used for analysis of OS.

Univariate and multivariate survival analyses with calculation of hazard ratios (HR) were performed using Cox’s proportional-hazards model. R software was used for statistical analysis and a *p*-value ≤ 0.05 was considered significant.

Regarding the correlation of CNA with mRNA expression, a gene-level table of copy number values and gene expression data were downloaded from the COAD-TCGA dataset. Using as input CNA and miRNA expression, we describe the relationship between these variables by linear regression analysis setting as significant Pearson correlation value >0.45 (*R*^2^ > 0.2, *p*-value ≤ 0.01)

### Quantitative real-time (Q-PCR)

To validate bioinformatic analysis of CN alterations we performed real-time PCR in selected genes. We used GAPDH to normalize the data and PBLs as reference samples. Reactions were performed using SYBR Green I PCR Master Mix (Thermofisher), which includes the internal reference (ROX). Each qPCR reaction comprised 10 μl 2× SYBR Green PCR Master Mix, forward and reverse primers at final concentration of 500 nM. QPCR reactions were performed using the Quantstudio 12 K Flex (Thermofisher). The reaction profile was: initial step, 50° C for 2 min, denaturation, 95° C for 10 min, then 40 cycles of denaturing at 95° C for 15 sec and combined annealing and extension at 60° C for 60 sec. Each qPCR experiment contained triplicates of the no-template-controls and test samples for all of the primers tested. Three independent experiments were conducted for each analysis. Statistical analysis was performed with one-way ANOVA and Dunnett’s multiple comparisons test using Graphpad software. ^*^p < 0.05, ^**^p < 0.01, ^***^p < 0.001 and ^****^p < 0.0001.

### Immunoistochemistry

Immunostaining was performed with standard protocols using Bond^™^ Polymer Refine Detection Kit (Leica Biosystem, Buffalo Grove, IL) according to the manufacturer’s instructions, on formalin-fixed, paraffin-embedded tissues. Sections (5 μm) were mounted on slides and stained with hematoxylin and eosin to be evaluated by light microscopy. The antibody used in immunostaining for CCND1 (#3642, DAKO, Carpinteria, CA, USA).

## SUPPLEMENTARY MATERIALS FIGURES AND TABLES
















