# Supplementary material for: Identification of copy number alterations in colon cancer from analysis of amplicon-based next generation sequencing data
Source: Oncotarget. 2018 Apr 17;9(29):20409–25. doi: 10.18632/oncotarget.24912 (PMC5945505; doi:10.18632/oncotarget.24912)
Supplement: Supplementary file 1 [file oncotarget-09-20409-s001.pdf]

Identification of copy number alterations in colon cancer from analysis of amplicon-based Next generation sequencing data

SUPPLEMENTARY MATERIALS

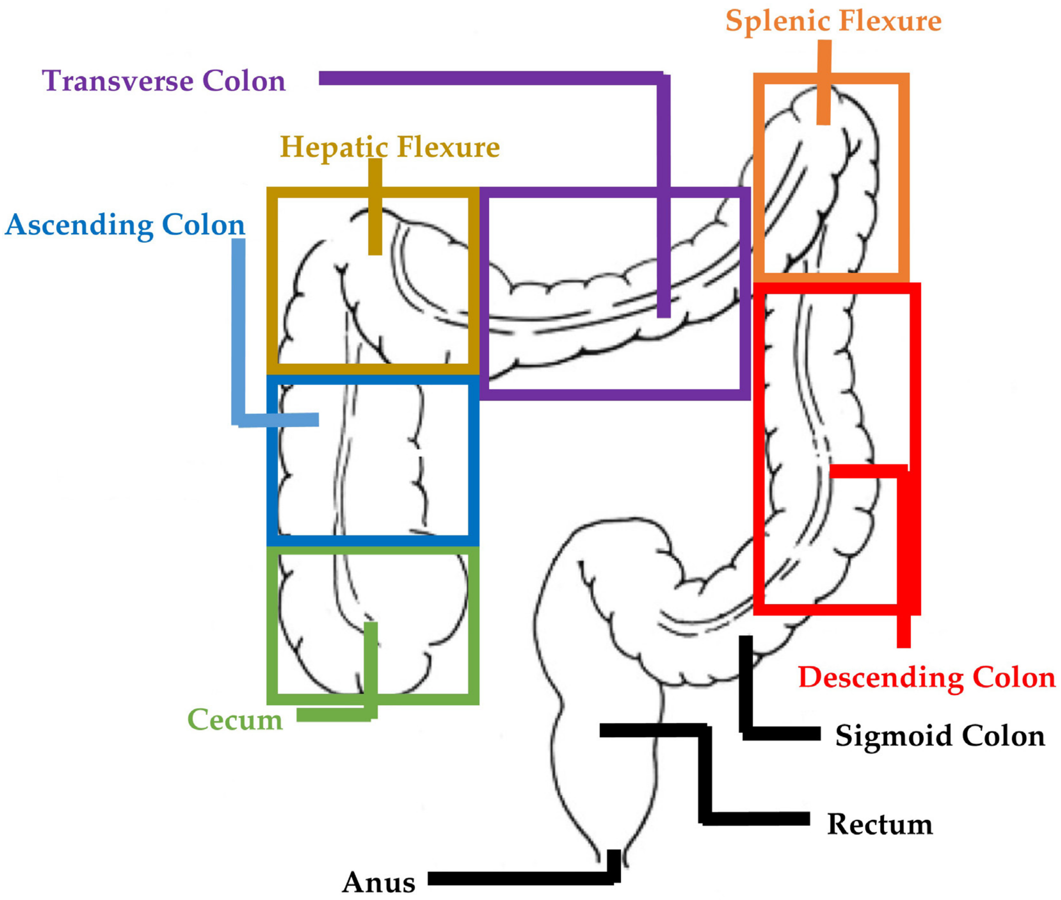

Supplementary Figure 1: Schematic representation of the different colon segments in which tumors under analysis were resected.

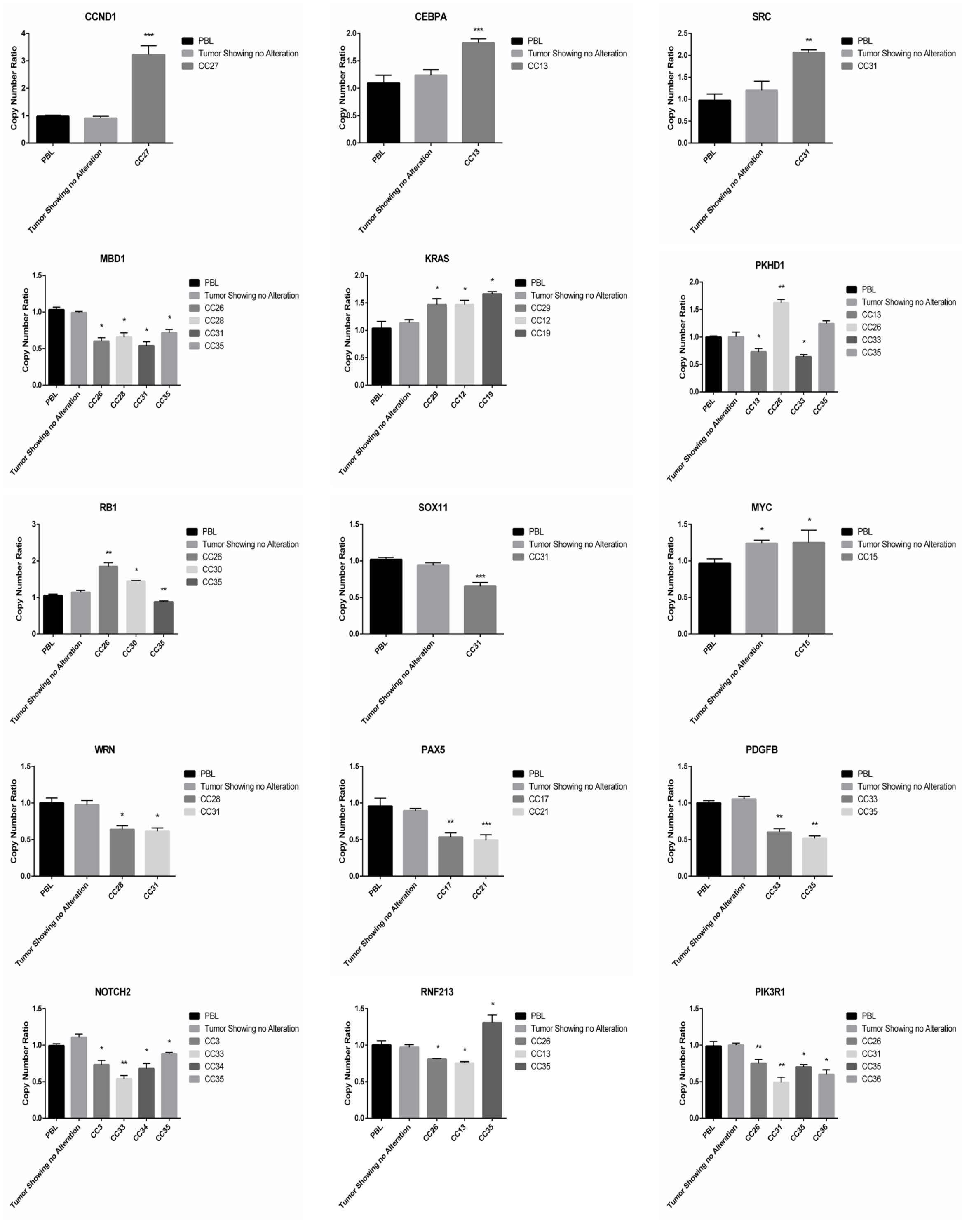

Supplementary Figure 2: Q-PCR analysis of several colon cancer samples presenting CN Gains in: CCND1, CEBPA, SRC, KRAS and c-MYC; CN losses in SOX11, PAX5, PIK3R1, MBD1, NOTCH2 and PDGFB or discording CNAs across different samples RB1, RNF213, WRN and PKHD1. Values are expressed as relative CN ratios using as standard the median value 3 PBL samples set as 1. Tumors presenting normal CN for the specific gene were also included in each analysis.

Cecum

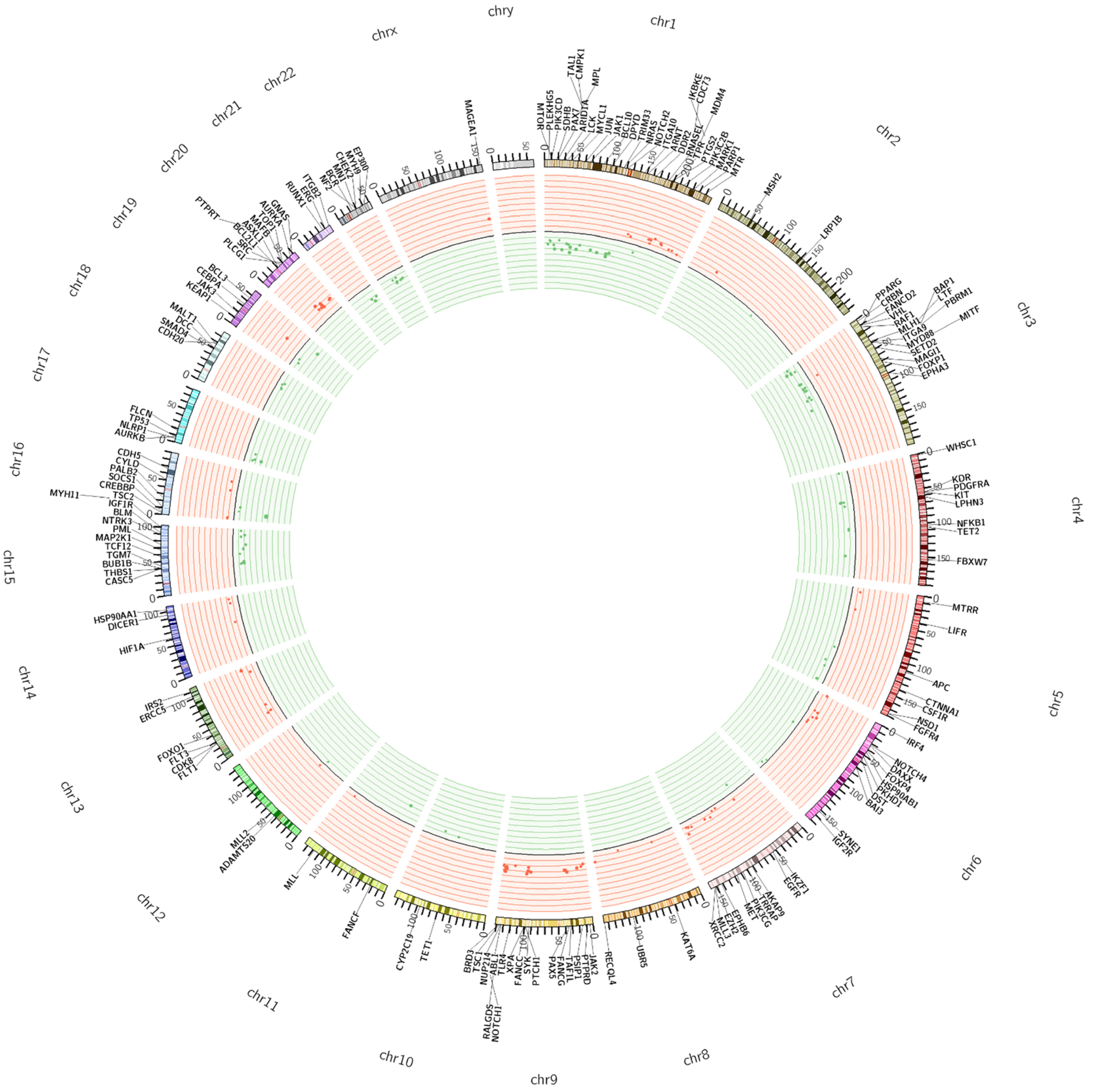

Supplementary Figure 3: The Circos plot summarizes all CNAs detected in colon cancers samples arising from cecum segment. The two outermost tracks report the distributions of 409 genes along the genome; the inner most tracks reported the values of the log2 CN ratio. Genes with altered CN are distinguished by colour as deletions (green) and amplifications (red).

Ascending Colon

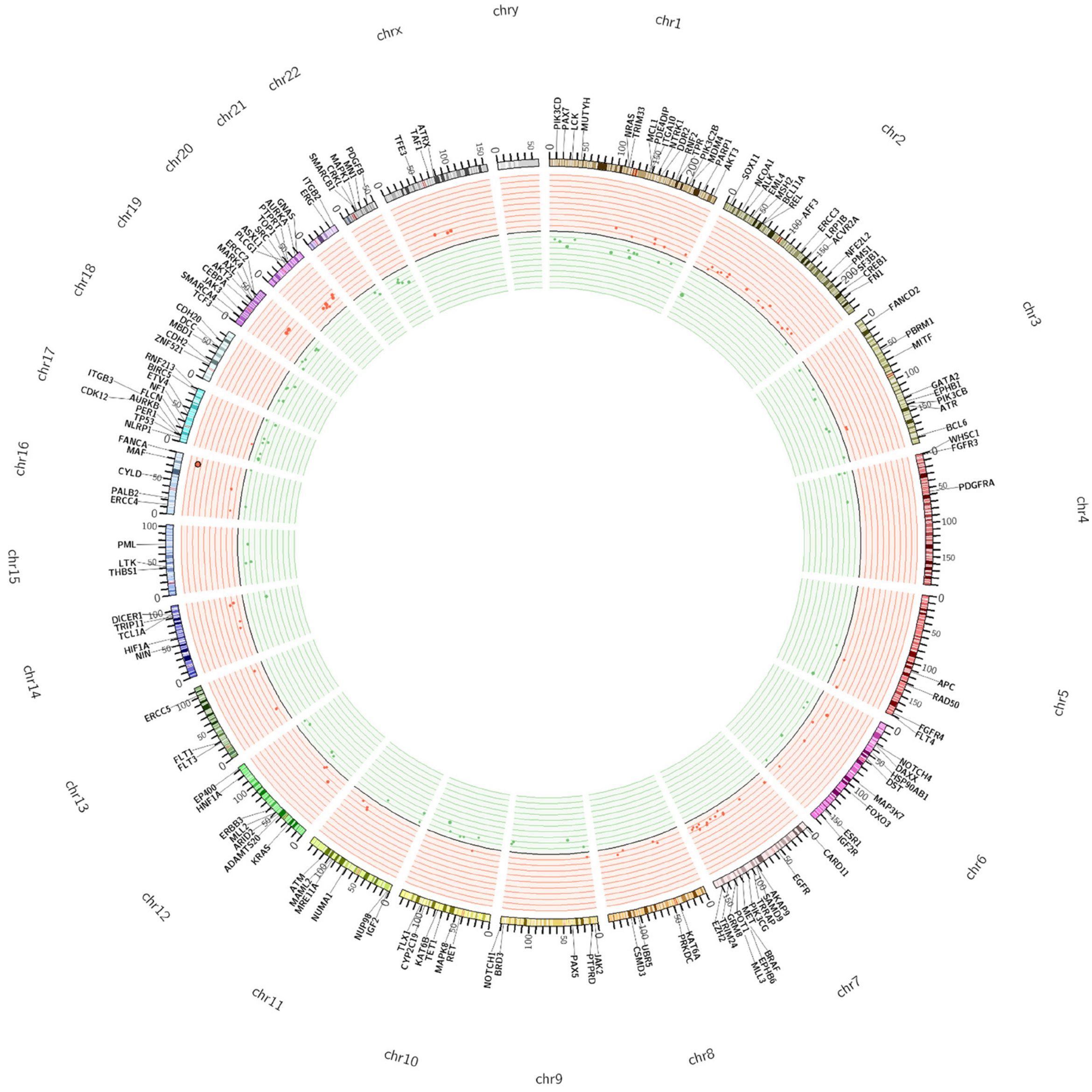

Supplementary Figure 4: The Circos plot summarizes all CNAs detected in colon cancers samples arising from ascending colon segment. The two outermost tracks report the distributions of 409 genes along the genome; the inner most tracks reported the values of the log2 CN ratio. Genes with altered CN are distinguished by colour as deletions (green) and amplifications (red).

Hepatic Flexure

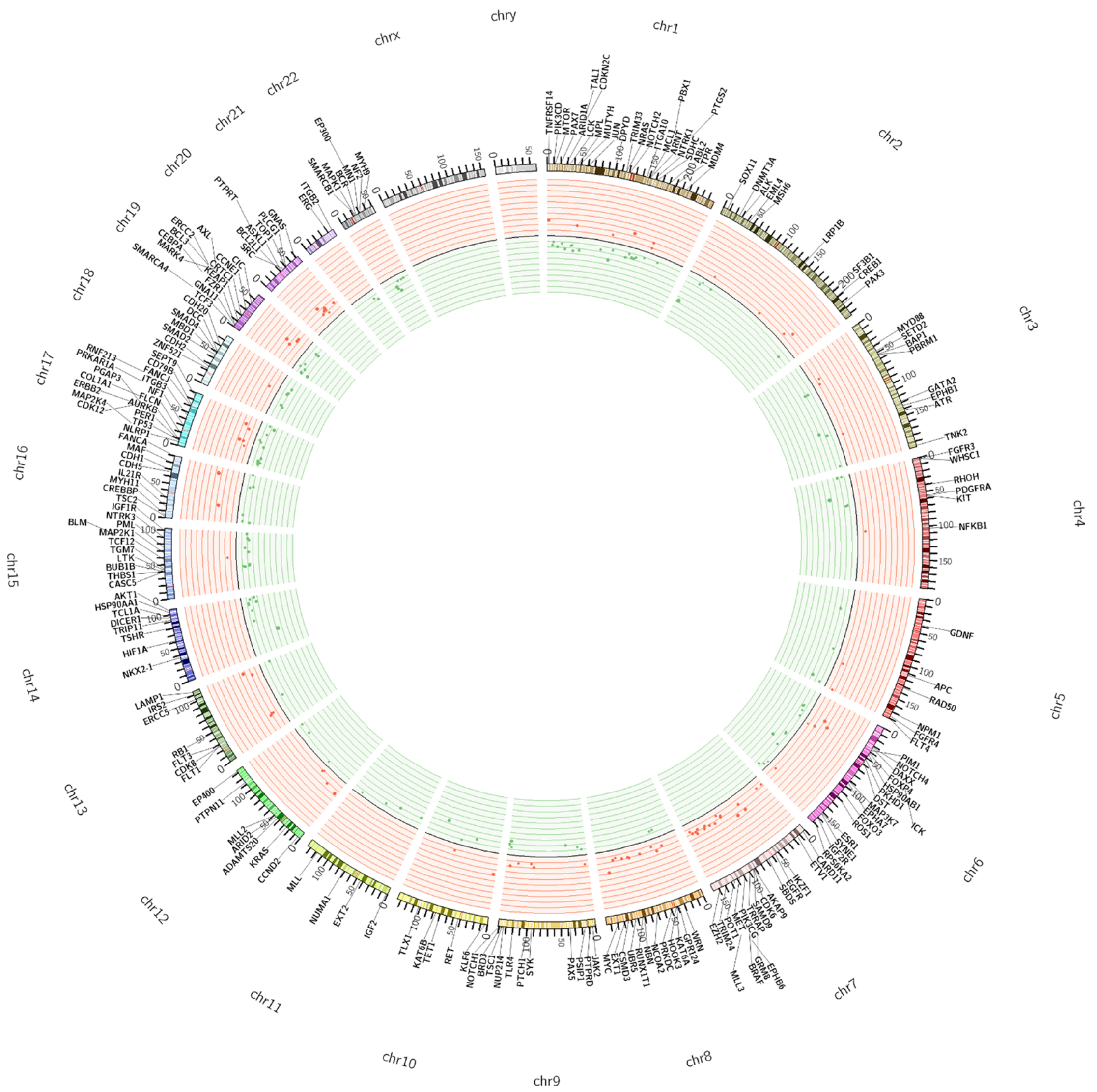

Supplementary Figure 5: The Circos plot summarizes all CNAs detected in colon cancers samples arising from hepatic flexure segment. The two outermost tracks report the distributions of 409 genes along the genome; the inner most tracks reported the values of the log2 CN ratio. Genes with altered CN are distinguished by colour as deletions (green) and amplifications (red).

Transverse Colon

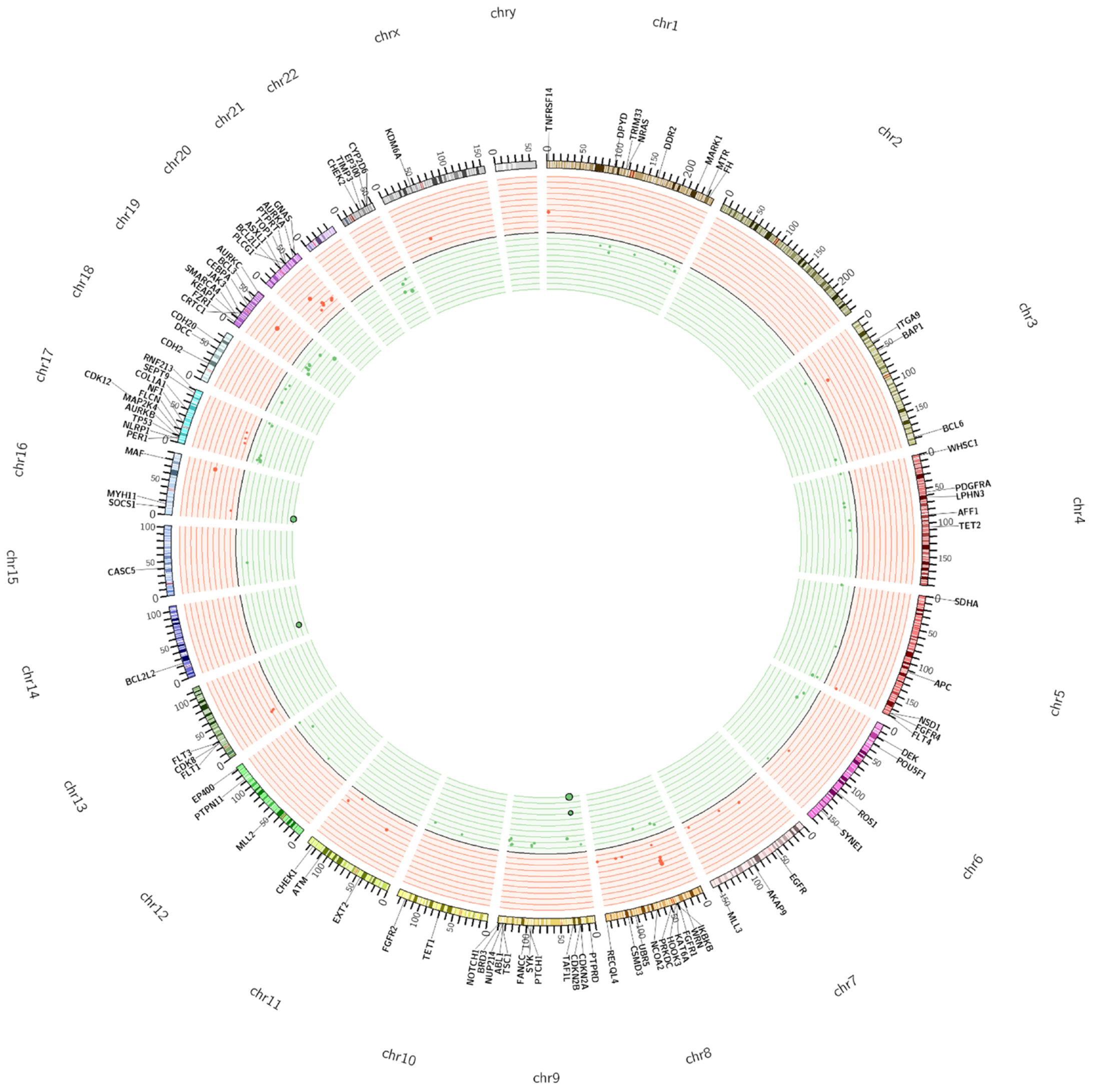

Supplementary Figure 6: The Circos plot summarizes all CNAs detected in colon cancers samples arising from transverse segment. The two outermost tracks report the distributions of 409 genes along the genome; the inner most tracks reported the values of the log2 CN ratio. Genes with altered CN are distinguished by colour as deletions (green) and amplifications (red).

Splenic Flexure

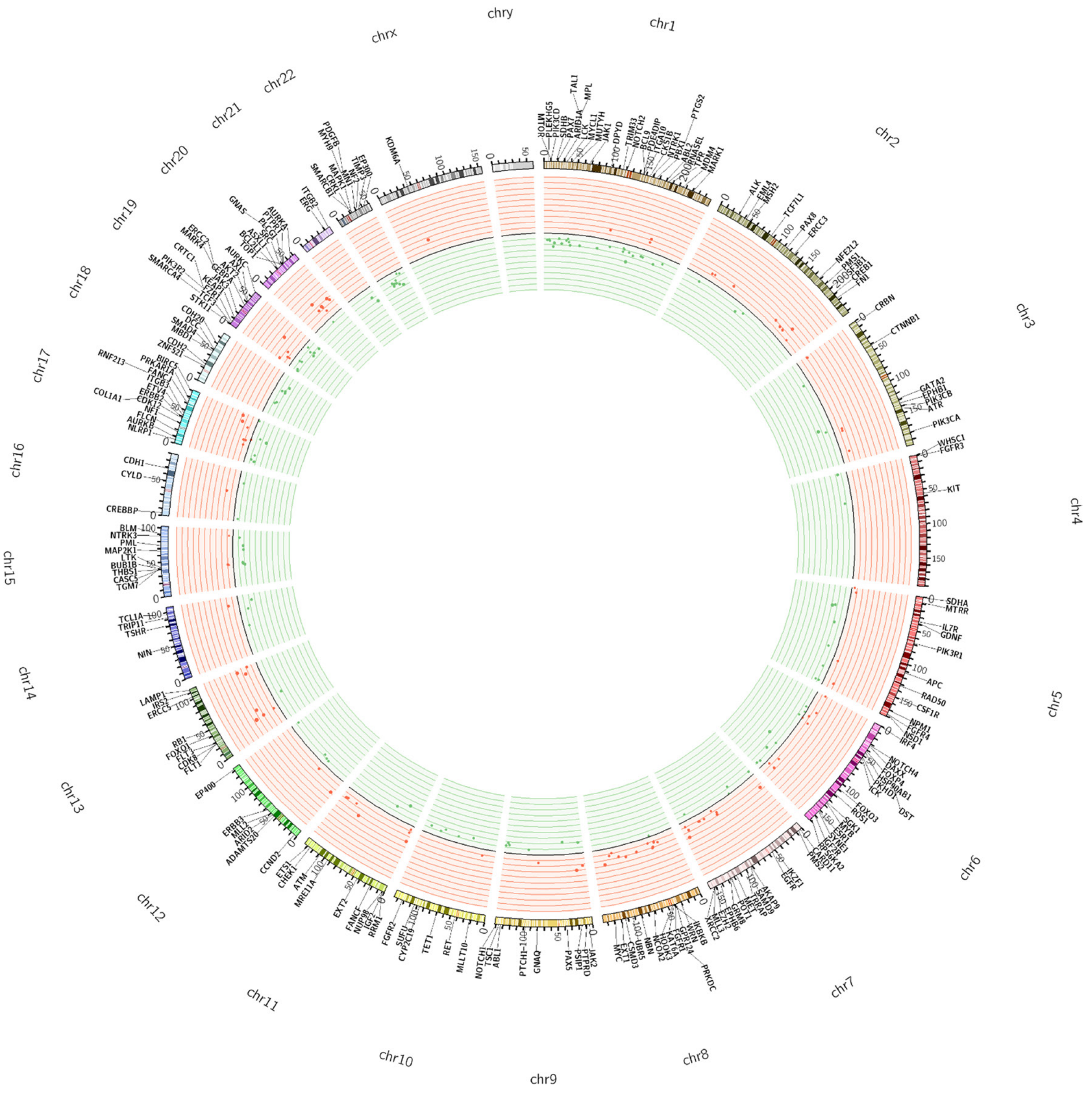

Supplementary Figure 7: The Circos plot summarizes all CNAs detected in colon cancers samples arising from splenic flexure segment. The two outermost tracks report the distributions of 409 genes along the genome; the inner most tracks reported the values of the log2 CN ratio. Genes with altered CN are distinguished by colour as deletions (green) and amplifications (red).

Descending Colon

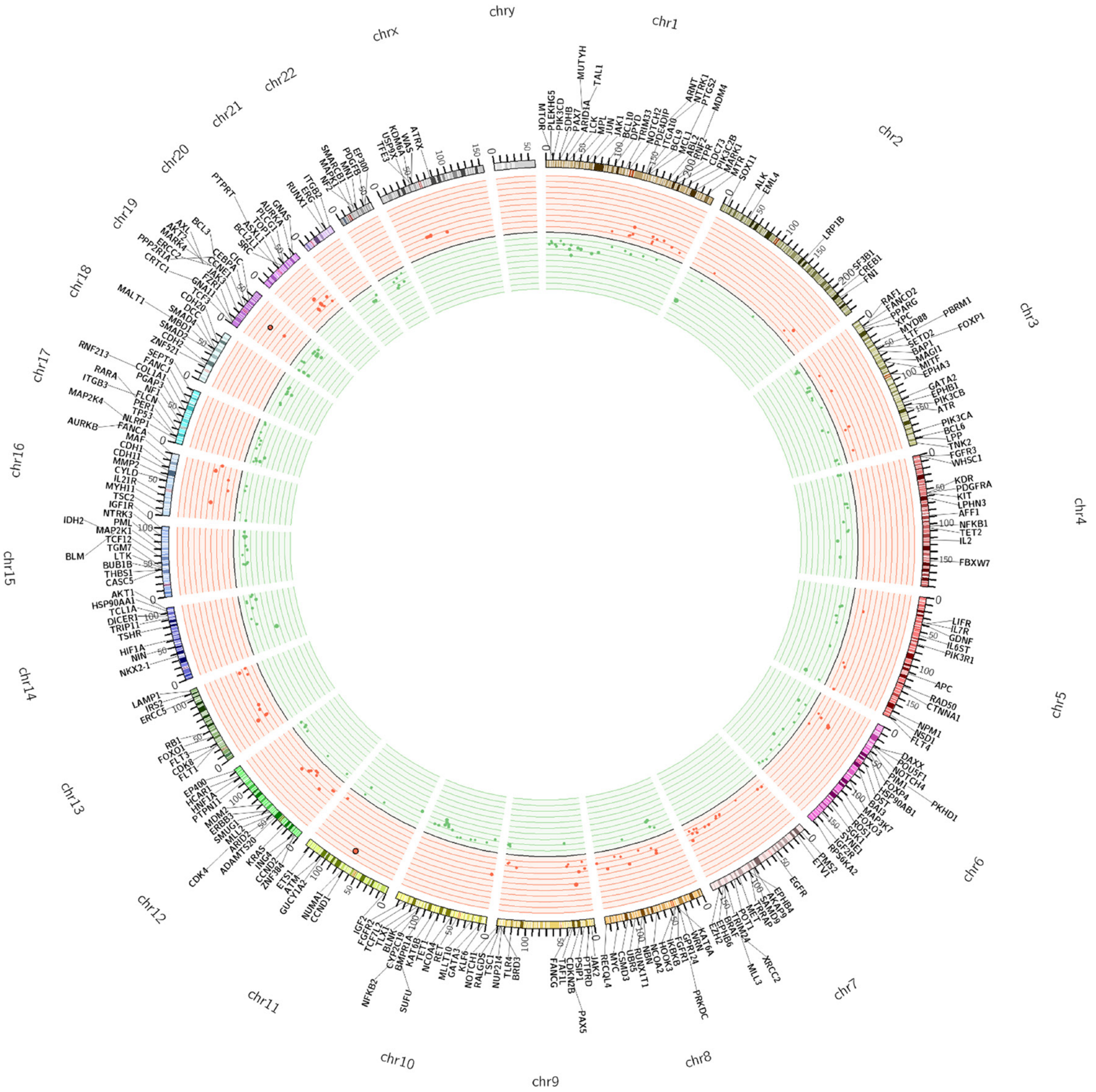

Supplementary Figure 8: The Circos plot summarizes all CNAs detected in colon cancers samples arising from descending colon segment. The two outermost tracks report the distributions of 409 genes along the genome; the inner most tracks reported the values of the log2 CN ratio. Genes with altered CN are distinguished by colour as deletions (green) and amplifications (red).

Supplementary Table 1: Clinical-pathological characteristics of patients under study. See Supplementary_Table_1

Supplementary Table 2: Genes showing significant CNAs based on Benjamini–Hochberg FDR selection. See Supplementary_Table_2

Supplementary Table 3: Genes showing significant CNAs ordered by cytoband. See Supplementary_Table_3

Supplementary Table 4: Correlation of CNAs with the mRNA expression profile of the corresponding genes in TCGA-COAD patients by linear regression analysis. See Supplementary_Table_4

Supplementary Table 5: Association between biomarkers and clinical-pathological parameters were assessed using contingency tables with a Fisher's and χ2 test. See Supplementary_Table_5

Supplementary Table 6: "Common genes" showing CNAs in tumors arising from all colon segments. See Supplementary_Table_6

Supplementary File 1: Genes showing CNAs identified by Amplicon CNA algorithm in 37 colon cancer segment. See Supplementary_File_1
